# Synthesis, Characterization, and Anti-Inflammatory Activity of Newer Quinazolinone Analogs

**DOI:** 10.1155/2013/907525

**Published:** 2013-01-17

**Authors:** Chatrasal Singh Rajput, Shiwani Singhal

**Affiliations:** ^1^Medicinal Chemistry Division, Department of Pharmacology, L.L.R.M. Medical College, Meerut 250002, India; ^2^Jubilant Chemsys Ltd. R & D B-34, Sector 58, Noida 201301, India

## Abstract

A series of 3-[2′-(Substitutedbenzylideneamino)phenyl]-2-methyl-6-substituted quinazolin-4-ones (**5–10**), 3-[2′-(3^″^-chloro-2^″^-oxo-4^″^-substitutedphenylazetidin-1^″^-yl)phenyl]-2-methyl-6-substitutedquinazolin-4-ones (**11–16**), and 3-[2′-(2^″^-substitutedphenyl-4^″^-oxo-1^″^,3^″^-thiazolidin-3^″^-yl)phentl]-2-methyl-6-substitutedquinazolin-4-ones (**17–22**) have been synthesized in the present study. The structures of the synthesized compounds were assigned on the basis of elemental analysis, IR, ^1^H NMR, and mass spectral data. All the newly synthesized compounds were screened for anti-inflammatory and analgesic activities.

## 1. Introduction

Quinazolinone derivatives represent one of the most active classes of compounds possessing a wide spectrum of biological activity. They are widely used in pharmaceuticals and agrochemicals. Several reports have been published on the biological activities of quinazolinone derivatives, including their anti-inflammatory [1–7], antimalarial [8, 9], antimicrobial, anti-fungal, antibacterial [10–16], anticonvulsant [17–20], and antitumor [21, 22] activities. Moreover, large number of quinazolinone derivatives having substitution at 2 and 3 position by different heterocyclic moieties increases anti-inflammatory potential of quinazolinone derivatives. Similarly, various azetidinones [23–25] and thiazolidinones [26, 27] have been reported to possess potent anti-inflammatory activity. Looking to the medicinal importance of 4(3*H*)-quinazolinone, 4-thiazolidinone, and azetidinones, we report here the synthesis of a new class of heterocyclic molecules in which all of these moieties are present and try to develop potential bioactive molecules. The structures of the compounds synthesized were assigned on the basis of elemental analysis, IR, ^1^H NMR, and Mass spectral data. These compounds were evaluated for their anti-inflammatory and analgesic activities.

## 2. Materials and Methods

### 2.1. Chemistry

**Scheme 1:**
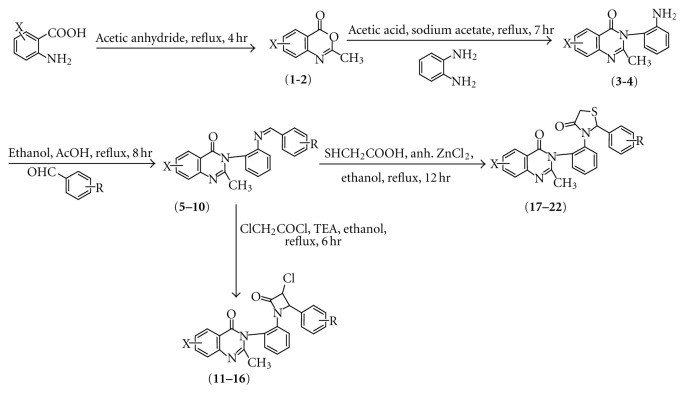


The synthetic routes of compounds are outlined in Scheme 1. As shown in Scheme 1, compounds 2-methylsubstitued-4H-3,1-benzoxazin-4-ones (1,2) were synthesized by the known procedure of Bogert [28]. Substituted anthranilic acid reacted with acetic anhydride to give 2-methylsubstituted-4H-3,1-benzoxazin-4-ones (1,2), which further reacted with 2-amino phenyl amine in acetic acid to yield 3-(2′-aminophenyl)-2-methyl-6-substituted quinazolin-4-ones (**3,4**). Compounds (**3,4**) when treated with different substituted aromatic aldehydes formed various substituted arylidene derivatives (**5–10**). These arylidene derivatives on treatment with chloroacetylchloride in presence of triethylamine yielded 3-[2′-(3′′-chloro-2′′-oxo-4′′-substituted phenylazetidin-1′′-yl)phenyl]-2-methyl-6-substitutedquinazolin-4-ones (**11–16**). On the other hand, these arylidene derivatives on reaction with thioglycolic acid and anhydrous ZnCl_2_ furnished 3-[2′-(2′′-substitutedphenyl-4′′-oxo-1′′,3′′-thiazolidin-3′′-yl)phenyl]-2-methyl-6-substitutedquinazolin-4-ones. (**17–22**). The structures of the compounds synthesized were assigned on the basis of elemental analysis, IR, ^1^H NMR, and Mass spectral data.

### 2.2. Methods

#### 2.2.1. Biological Activity

The experiments were performed with albino rats of the Charles-foster stain of either sex, excluding pregnant females, of 70–95 d. weighting 100–150 g. Food (chaw pallet) and water were given to the animals ad libitum. All the compounds were dissolved in propylene glycol. Phenylbutazone was used as reference drug.

*Anti-Inflammatory Activity against Carrageenan-Induced Rat's Paw Oedema*

This study was done with the procedure of Winter et al. [29]. The rats were divided into three groups (control, treated, and standard drug) of six animals each. A freshly prepared suspension of carrageenan (1% in 0.9% saline) 0.05 mn was injected under the planter aponeurosis of the right hind paw of each rat. Test compounds and standard drug were administered orally to the animals of drug-treated groups and the standard drug group, respectively, 1 h before the carrageenan injection. The paw volume of each rat was measured before 1 and after 3 h of carrageenan treatment with the help of a plethysmometer. The percent anti-inflammatory activity was calculated according to the formula given below

(1) $$\begin{array}{c} \text{Percentage}\;\text{of}\;\text{inhibition}\;\text{of}\;\text{oedema}=\left(1-\frac{V_{t}}{V_{c}}\right)\times 100, \end{array}$$

where *V*_*t*_ and *V*_*c*_ are the mean increase in paw volume of rats of the treated and the control group, respectively. Results were statistically analyzed.

*Analgesic Activity*

This activity was assessed following the method of Berkowitz et al. [30]. This method is based on the property of the test compound to antagonize the phenyl quinone-induced pain syndrome in mice. Groups of five mice were injected intraperitoneally with 0.25 mL of a 0.02% solution of phenylquinone in ethanol (5%) 1 h after of oral administration of the test compound. The number of writhes induced in each mouse was counted for 5 min (between 5 and 10 min) after injection of an irritant. The analgesic effect was expressed as percent protection in comparison to control.

(2) %  protection=(1−mean  number  of  writhes  in  mice  of  test  groupsmean  number  of  writhes  in  mice  of  control  group  ) ×100.

#### 2.2.2. Experimental

Melting points were determined in one-end-open capillary tube on Thomas Hoover melting apparatus and are uncorrected. The purities of compounds were checked by thin layer chromatography (TLC) using silica gel-G coated plats. The spots were developed in an iodine chamber and visualized under ultraviolet (UV) lamp. Infrared (IR) absorption spectra were recorded on Perkin-Elmer RX-1 FTIR spectrometer using potassium bromide (KBr) pellet, and wave numbers were given in cm^−1^. The ^1^H NMR spectra were recorded in CDCl_3_ on a Bruker Avance II 400 NMR spectrometer (400 MHz). The ^13^C NMR spectra were recorded in CDCl_3_ on a Bruker Avance II 400 NMR spectrometer operating at 100 MHz. The chemical shifts are reported in part per million (*δ* ppm) using trimethylsilane (TMS) as an internal standard. Elemental analysis (C, H, N) was performed on Perkin-Elmer 2400 analyzer, and values were either in ±0.4% of the calculated. 

*3-(*2′*-Aminophenyl)-2-Methyl-Quinazolin-4-one (* ***3***)

A mixture of 2-methyl-4H-3,1-benzoxazin-4-one (1) (0.01 mole) with 2-aminophenylamine (0.01 mole) in acetic acid (40 mL) was refluxed for 7 hr. in the presence of sodium acetate. The solvent was removed under reduced pressure. The solid thus obtained was filtered off and recrystallized from toluene to give compound **3** (78%). m.p. 99°C. IR (KBr) *ν*_max_ in cm^−1^: 3033 (CH aromatic), 2920 (CH aliphatic), 1690 (C=O), 1595 (C=N); ^1^HNMR (CDCl_3_) *δ* in ppm: 9.10 (s, 2H, NH_2_ exchangeable), 6.89–7.61 (m, 8H, Ar–H), 2.29 (s, 3H, CH_3_); Anal. Calcd. for C_15_H_13_N_3_O: C, 71.71; H, 5.18; N, 16.73: Found: C, 71.85; H, 5.20; N, 16.68: MS [M]^+^ at *m/z* 251.

The following compound was prepared using a similar procedure described for compound **3**. 

*3-(*2′*-Aminophenyl)-2-Methyl-6-Bromoquinazolin-4-one (* ***4***)

(75%) (Benzene) m.p. 199°C, IR (KBr) *ν*_max_ in cm^−1^: 3030 (CH aromatic), 2925 (CH aliphatic), 1695 (C=O), 1598 (C=N); ^1^H NMR (CDCl_3_) *δ* in ppm: 9.06 (s, 2H, NH_2_ exchangeable), 6.85–7.51 (m, 7H, Ar–H), 2.24 (s, 3H, CH_3_); Anal. Calcd. for C_15_H_12_N_3_OBr: C, 54.55; H, 3.64; N, 12.73: Found: C, 54.38; H, 3.65; N, 12.76: MS [M]^+^ at *m/z* 330.

*3-[* 2′*-(Benzylideneamino)Phenyl]-2-Methyl-Quinazolin-4-one (* ***5***)

A solution of compound** 3** (0.01 moles) in ethanol (55 mL) was refluxed with benzaldehyde (0.01 moles) separately in the presence of glacial acetic acid (2.5 mL) for 11 hr. The reaction mixture was concentrated, cooled, and then poured into ice water. The separated solid was filtered and recrystallized from methanol to give compound **29** (63%) m.p. 118°C: IR (KBr) *ν*_max_ in cm^−1^: 3028 (CH aromatic), 2930CH aliphatic), 1705 (C=O), 1610 (C=N); ^1^HNMR (CDCl_3_) *δ* in ppm: 6.79–7.63 (m, 13H, Ar–H), 6.35 (s, 1H, N=CH), 2.21 (s, 3H, CH_3_); Anal. Caled. for C_22_H_17_N_3_O: C, 77.88; H, 5.01; N, 12.39: Found: C, 77.96; H, 5.00; N, 12.41: MS [M]^+^ at *m/z*. 339.

The following compounds were prepared using a similar procedure described for compound **5**.

*3-[*2′*-(p-Chlorobenzylideneamino)Phenyl]-2-Methyl-Quinazolin-4-one (* ***6***)

(65%). (Benzene) m.p. 134°C: IR (KBr) *ν*_max_ in cm^−1^: 3028 (CH aromatic), 2929 (CH aliphatic), 1705 (C=O), 1598 (C=N) 788 (C–Cl); ^1^H NMR (CDCl_3_) *δ* in ppm: 6.82–7.65 (m, 12H, Ar–H), 6.38 (s, 1H, N=CH), 2.24 (s, 3H, CH_3_); Anal. Caled. for C_22_H_16_N_3_OCl: C,70.68; H, 4.28; N, 11.24: Found: C, 70.79; H, 4.27; N, 11.26: MS [M]^+^ at *m/z*. 373.5.

*3-[*2′*-(p-Methoxybenzylideneamino)Phenyl]-2-Methyl-Quinazolin-4-one (* ***7***)

(60%). (Methanol) m.p. 148°C: IR (KBr) *ν*_max_ in cm^−1^: 3035(CH aromatic), 2932 (CH aliphatic), 1700 (C=O), 1600 (C=N); ^1^HNMR (CDCl_3_) *δ* in ppm: 6.80–7.61 (m, 12H, Ar–H), 6.40 (s, 1H, N=CH), 3.48 (s, 3H, OCH_3_) 2.26 (s, 3H, CH_3_); Anal. Caled. for C_23_H_19_N_3_O_2_: C, 74.80; H, 5.15; N, 11.38: Found: C, 74.96; H, 5.17; N, 11.29: MS [M]^+^ at *m/z*. 369.

*3-[* 2′*-(Benzylideneamino)Phenyl]-2-Methyl-6-Bromoquinazolin-4-one (* ***8***)

(64%). (Ethanol) m.p. 215°C: IR (KBr) *ν*_max_ in cm^−1^: 3033 (CH aromatic), 2926 (CH aliphatic), 1698 (C=O), 1606 (C=N); ^1^H NMR (CDCl_3_) *δ* in ppm: 6.83–7.60 (m, 12H, Ar–H), 6.43 (s, 1H, N=CH), 2.22 (s, 3H, CH_3_); Anal. Caled. for C_22_H_16_N_3_OBr: C,63.16; H, 3.83; N, 10.05: Found: C, 63.29; H, 3.82; N, 10.07: MS [M]^+^ at *m/z*. 418.

*3-[* 2′*-(p-Chlorobenzylideneamino)Phenyl]-2-Methyl-6-Bromoquinazolin-4-one (* ***9***)

(65%). (Ethanol) m.p. 234°C: IR (KBr) *ν*_max_ in cm^−1^: 3030 (CH aromatic), 2930 (CH aliphatic), 1695 (C=O), 1601 (C=N), 785 (C–Cl); ^1^HNMR (CDCl_3_) *δ* in ppm: 6.81–7.59 (m, 11H, Ar–H), 6.41 (s, 1H, N=CH), 2.25 (s, 3H, CH_3_); Anal. Caled. for C_22_H_15_N_3_OBrCl: C, 58.34; H, 3.31; N, 9.28: Found: C, 58.55; H, 3.32; N, 9.30: MS [M]^+^ at *m/z*. 452.5.

*3-[*2′*-(p-Methoxybenzylideneamino)Phenyl]-2-Methyl-6-Bromoquinazolin-4-one (* ***10***)

(60%). (Acetone) m.p. 223°C: IR (KBr) *ν*_max_ in cm^−1^: 3033 (CH aromatic), 2926 (CH aliphatic), 1698 (C=O), 1595 (C=N); ^1^HNMR (CDCl_3_) *δ* in ppm: 6.83–7.61 (m, 11H, Ar–H), 6.44 (s, 1H, N=CH), 3.44 (s, 3H, OCH_3_) 2.25 (s, 3H, CH_3_); Anal. Caled. for C_23_H_18_N_3_O_2_Br: C, 61.61; H, 4.02; N, 9.38: Found: C, 61.52; H, 4.01; N, 9.36: MS [M]^+^ at *m/z*. 448

*3-[*2′*-(*3′′*-Chloro- *2′′*-Oxo- *4′′*-Phenylazetidin- *1′′*-Yl)-Phenyl]-2-Methyl-Quinazol-In-4-one (* ***11***)

To a solution of compound **5** (0.01 mole) and triethylamine (0.5 mL) in ethanol (55 mL) was added in monochloroacetylchloride (0.014 mole) at 50°C. The reaction mixture was stirred for 45 min. at room temperature and refluxed for 8 hr. The reaction mixture was filtered to removed triethylamine hydrogen chloride and the resultant solution was poured onto crushed ice with constant stirring. The solid thus obtained was recrystallized from acetone to give desired compound **11** (60%) m.p. 153°C. IR (KBr): *ν*_max_ in cm^−1^: IR (KBr) *ν*_max_ in cm^−1^: 3030 (CH aromatic), 2925 (CH aliphatic), 1700 (C=O), 1595 (C=N); ^1^HNMR (CDCl_3_) *δ* in ppm: 6.81–7.98 (m, 13H, Ar–H), 6.51 (d,1H, J = 9.0 Hz, CH–Cl), 4.60 (d, 1H, J = 9.3 Hz, N–CH–Ar), 2.21 (s, 3H, CH_3_);. Anal. Calcd. for C_24_H_18_N_3_O_2_Cl: C, 69.31; H, 4.33; N, 10.11: Found: C, 69.47; H, 4.32; N, 10.14: MS [M]^+^at *m/z* 415.5.

The following compounds were prepared using a similar procedure described for compound **11**. 

*3-[*2′*-(*3′′*-Chloro- *2′′*-Oxo- *4′′*-(p-Chlorophenyl)Azetidin- *1′′*-yl)-Phenyl]-2-Methyl Quinazolin-4-one (* ***12***)

(58%) (Methanol) m.p. 198°C. IR (KBr): *ν*_max_ in cm^−1^: IR (KBr) *ν*_max_ in cm^−1^: 3033 (CH aromatic), 2928 (CH aliphatic), 1705 (C=O), 1591 (C=N), 788 (C–Cl); ^1^HNMR (CDCl_3_) *δ* in ppm: 6.83–7.95 (m, 12H, Ar–H), 6.56 (d,1H, J = 8.8 Hz, CH–Cl), 4.62 (d, 1H, J = 9.0 Hz, N–CH–Ar), 2.25 (s, 3H, CH_3_);. Anal. Calcd. for C_24_H_17_N_3_O_2_Cl_2_: C, 64.00; H, 3.78; N, 9.33: Found: C, 64.15; H, 3.80; N, 9.30: MS [M]^+^at *m/z* 450.

*3-[*2′*-(*3′′*-Chloro- *2′′*-Oxo- *4′′*-(p-Methoxyphenyl)Azetidin- *1′′*-yl)-Phenyl]-2-Methyl Quinazolin-4-one (* ***13***)

(56%) (Ethanol)m.p. 212°C. IR (KBr): *ν*_max_ in cm^−1^: IR (KBr) *ν*_max_ in cm^−1^ : 3028 (CH aromatic), 2930 (CH aliphatic), 1700 (C=O), 1595 (C=N); ^1^HNMR (CDCl_3_) *δ* in ppm: 6.81–7.93 (m, 12H, Ar–H), 6.54 (d,1H, J = 9.0 Hz, CH–Cl), 4.65 (d, 1H, J = 9.3 Hz, N–CH–Ar), 3.47 (s, 3H, OCH_3_) 2.23 (s, 3H, CH_3_);. Anal. Calcd. for C_25_H_20_N_3_O_3_Cl: C, 67.34; H, 4.49; N, 9.43: Found: C, 67.15; H, 4.50; N, 9.40: MS [M]^+^at *m/z* 445.5.

*3-[* 2′*-(*3′′*-Chloro-*2′′*-Oxo-*4′′*-Phenylazetidin-*1′′*-yl)-Phenyl]-2-Methyl-6-Bromo-Quinazolin-4-one (* ***14***)

(58%) (Methanol) m.p. 176°C. IR (KBr): *ν*_max_ in cm^−1^: IR (KBr) *ν*_max_ in cm^−1^ : 3030 (CH aromatic), 2925 (CH aliphatic), 1695 (C=O), 1595 (C=N); ^1^HNMR (CDCl_3_) *δ* in ppm: 6.80–7.92 (m, 12H, Ar–H), 6.54 (d, 1H, J = 9.2 Hz, CH–Cl), 4.64 (d, 1H, J = 9.1 Hz, N–CH–Ar), 2.22 (s, 3H, CH_3_);. Anal. Calcd. for C_24_H_17_N_3_O_2_ClBr: C, 58.24; H, 3.44; N, 8.49: Found: C, 58.36; H, 3.43; N, 8.51: MS [M]^+^at *m/z* 494.5.

*3-[* 2′*-(*3′′*-Chloro-*2′′*-Oxo-*4′′*-(p-Chlorophenyl)Azetidin-*1′′*-yl)-Phenyl]-2-Methyl-6-Bromo Quinazolin-4-one (* ***15***)

(54%) (Ethanol) m.p. 204°C. IR (KBr): *ν*_max_ in cm^−1^: IR (KBr) *ν*_max_ in cm^−1^: 3027 (CH aromatic), 2930 (CH aliphatic), 1710 (C=O), 1598 (C=N), 785 (C–Cl); ^1^HNMR (CDCl_3_) *δ* in ppm: 6.79–7.90 (m, 11H, Ar–H), 6.55 (d, 1H, J = 9.3 Hz, CH–Cl), 4.61 (d, 1H, J = 8.8 Hz, N–CH–Ar), 2.20 (s, 3H, CH_3_);. Anal. Calcd. for C_24_H_16_N_3_O_2_Cl_2_Br: C, 54.44; H, 3.02; N, 7.94: Found: C, 54.61; H, 3.01; N, 7.95: MS [M]^+^at *m/z* 529.

*3-[* 2′*-(*3′′*-Chloro-*2′′*-Oxo-*4′′*-(P-Methoxyphenyl)Azetidin-*1′′*-yl)-Phenyl]-2-Methyl Quinazolin-4-one (* ***16***)

(60%) (Acetone) m.p. 246°C. IR (KBr): *ν*_max_ in cm^−1^: IR (KBr) *ν*_max_ in cm^−1^: 3030 (CH aromatic), 2930 (CH aliphatic), 1700 (C=O), 1590 (C=N); ^1^HNMR (CDCl_3_) *δ* in ppm: 6.80–7.92 (m, 11H, Ar–H), 6.52 (d, 1H, J = 9.2 Hz, CH–Cl), 4.62 (d, 1H, J = 9.0 Hz, N–CH–Ar), 3.49 (s, 3H, OCH_3_) 2.25 (s, 3H, CH_3_);. Anal. Calcd. for C_25_H_19_N_3_O_3_ClBr: C, 57.20; H, 3.62; N, 8.01: Found: C, 57.31; H, 3.61; N, 8.06: MS [M]^+^at *m/z* 524.5.

*3-[* 2′*-(*2′′*-Phenyl-*4′′*-Oxo-*1′′,3′′*-Thiazolidin-*3′′*-yl)Phenyl]-2-Methyl-6-Quinazolin-4-one (* ***17***)

A mixture of compound 5(0.01 moles), thioglycolic acid (0.012 moles) in absolute dioxane (75 mL) and in the presence of anhydrous ZnCl_2_ was refluxed for 18 hr. The solvent was removed under reduced pressure. The solid thus obtained was filtered and washed with water. The product finally obtained was dried and recrystallized from ethanol to yield compound **17** (55%) m.p. 164°C. IR (KBr): *ν*_max_ in cm^−1^: IR (KBr) *ν*_max_ in cm^−1 : ^3032 (CH aromatic), 2931 (CH aliphatic), 1705 (C=O), 1595 (C=N),685 (C–S–C); ^1^HNMR (CDCl_3_) *δ* in ppm: 6.80–7.97 (m, 13H, Ar–H), 4.55 (s, 1H, N–CH–Ar), 4.25 (s, 2H, CH_2_) 2.25 (s, 3H, CH_3_);. Anal. Calcd. for C_24_H_19_N_3_O_2_S: C, 69.73; H, 4.60; N, 10.17: Found: C, 69.82; H, 4.61; N, 10.22: MS [M]^+^at *m/z* 413.

*3-[* 2′*-(*2′′*-(p-Chlorophenyl)-*4′′*-Oxo-*1′′,3′′*-Thiazolidin-*3′′*-Yl)Phenyl]-2-Methyl-Quinazolin-4-one. (* ***18***)

(49%) (Methanol) m.p. 192°C. IR (KBr): *ν*_max_ in cm^−1^: IR (KBr) *ν*_max_ in cm^−1^ : 3030 (CH aromatic), 2930 (CH aliphatic), 1700 (C=O), 1598 (C=N), 784 (C–Cl), 688 (C–S–C); ^1^HNMR (CDCl_3_) *δ* in ppm: 6.83–7.96 (m, 12H, Ar–H), 4.58 (s, 1H, N–CH–Ar), 4.21 (s, 2H, CH_2_) 2.22 (s, 3H, CH_3_);. Anal. Calcd. for C_24_H_18_N_3_O_2_SCl: C, 64.36; H, 4.02; N, 9.39: Found: C,64.41; H, 4.03; N, 9.35: MS [M]^+^at *m/z* 447.5.

*3-[*2′*-(*2′′*-(p-Methoxyphenyl)- *4′′*-Oxo- *1′′, 3′′*-Thiazolidin- *3′′*-yl)Phenyl]-2-Methyl Quinazolin-4-one (* ***19***)

(50%) (Acetone) m.p. 204°C. IR (KBr): *ν*_max_ in cm^−1^: IR (KBr) *ν*_max_ in cm^−1^ : 3032 (CH aromatic), 2927 (CH aliphatic), 1700 (C=O), 1600 (C=N), 690 (C–S–C),; ^1^HNMR (CDCl_3_) *δ* in ppm: 6.81–7.95 (m, 12H, Ar–H), 4.52 (s, 1H, N–CHvAr), 4.23 (s, 2H, CH_2_) 3.51 (s, 3H, OCH_3_), 2.20 (s, 3H, CH_3_);. Anal. Calcd. for C_25_H_21_N_3_O_3_S: C, 67.72; H, 4.74; N, 9.48: Found: C,67.88; H, 4.73; N, 9.51: MS [M]^+^at *m/z* 443.

*3-[* 2′*-(*2′′*-Phenyl-*4′′*-Oxo-1*′′,3′′*-Thiazolidin-*3′′*-Yl)Phenyl]-2-Methyl-6-Bromo Quinazolin-4-one (* ***20***)

(55%) (Ethanol) m.p. 236°C. IR (KBr): *ν*_max_ in cm^−1^: IR (KBr) *ν*_max_ in cm^−1^: 3028 (CH aromatic), 2933 (CH aliphatic), 1705 (C=O), 1600 (C=N), 690 (C–S–C); ^1^H NMR (CDCl_3_) *δ* in ppm: 6.81–7.94 (m, 12H, Ar–H), 4.56 (s, 1H, N–CH–Ar), 4.22 (s, 2H, CH_2_) 2.24 (s, 3H, CH_3_). Anal. Calcd. for C_24_H_18_N_3_O_2_SBr: C, 58.54; H, 3.66; N, 8.54: Found: C,58.66; H, 3.67; N, 8.56: MS [M]^+^at *m/z* 492.5.

*3-[*2′*-(*2′′*-(p-Chlorophenyl)- *4′′*-Oxo-1*′′, 3′′*-Thiazolidin- *3′′*-yl)Phenyl]-2-Methyl-6-Bromo Quinazolin-4-one (* ***21***)

(48%) (Methanol) m.p. 267°C. IR (KBr): *ν*_max_ in cm^−1^: IR (KBr) *ν*_max_ in cm^−1^: 3033 (CH aromatic), 2925 (CH aliphatic), 1705 (C=O), 1595 (C=N), 784 (C–Cl), 685 (C–S–C); ^1^HNMR (CDCl_3_) *δ* in ppm: 6.80–7.93 (m, 11H, Ar–H), 4.52 (s, 1H, N–CH–Ar), 4.24 (s, 2H, CH_2_) 2.26 (s, 3H, CH_3_);. Anal. Calcd. for C_24_H_17_N_3_O_2_SClBr: C,54.70; H, 3.23; N, 7.98: Found: C, 54.86; H, 3.22; N, 7.97: MS [M]^+^at *m/z* 526.5.

*3-[*2′*-(*2′′*-(p-Methoxyphenyl)-4*′′*-Oxo-1*′′*,3*′′*-Thiazolidin-3*′′*-yl)Phenyl]-2-Methyl-6-Bromo Quinazolin-4-one (* ***22***)

(55%) (Ethanol) m.p. 254°C. IR (KBr): *ν*_max_ in cm^−1^: IR (KBr) *ν*_max_ in cm^−1^: 3031 (CH aromatic), 2928 (CH aliphatic), 1710 (C=O), 1605 (C=N), 685 (C–S–C); ^1^HNMR (CDCl_3_) *δ* in ppm: 6.81–7.92 (m, 11H, Ar–H), 4.59 (s,1H, N–CH–Ar), 4.25 (s, 2H, CH_2_), 3.49 (s, 3H, OCH_3_), 2.26 (s, 3H, CH_3_);. Anal. Calcd. for C_25_H_20_N_3_O_3_SBr: C, 57.47; H, 3.83; N, 8.05: Found: C, 57.62; H, 3.82; N, 8.03: MS [M]^+^at *m/z* 522.

## 3. Results and Discussion

### 3.1. Anti-Inflammatory Activity

The anti-inflammatory activity of newly synthesis compounds is showed in Table 1. All the compounds (**5–22**) were screened for anti-inflammatory activity at 50 mg/kg p.o. using phenylbutazone as reference drug. All the tested compounds exhibited 15.1 to 32.5% edema inhibition at the dose of 50 mg/kg p.o. When the compounds were substituted with *p*-chlorophenyl group showed better anti-inflammatory activity than phenyl group. Compounds (**5–10**) showed anti-inflammatory from 15.1 to 20.4%. Compound 9, which was substituted by 2′-(p-chlorobenzylideneamino)phenyl group at the IIIrd position of quinazolinone moiety showed better anti-inflammatory activity (20.4%) than compounds **5**, **6**, **7**, **8**, and 10. Cyclizations of these compounds into azetidinones and thiazolidinones have shown better anti-inflammatory activity than corresponding arylidene derivatives. Azetidinones (**11–16**) and thiazolidinones (**17–22**) have shown the anti-inflammatory activity from (24.6–27.3%) and (22.9–32.5%) respectively. In azetidinones, compound **15** showed better anti-inflammatory activity at the dose of 50 mg/kg p.o. than compounds **11**, **12**, **13**, **14**, and **16**. Compound **11** exhibited lower percentage inhibition of oedema (24.6%) among azetidinones. Thiazolidinones (**17–22**) generally showed better anti-inflammatory then corresponding azetidinones (**11–16**). Among all the tested compounds, 3-[2′-(2′′-(p-chlorophenyl)-4′′-oxo-1′′,3′′-thiazolidin-3′′-yl)phenyl]-2-methyl-6-bromo quinazolin-4-one (**21**) showed better anti-inflammatory activity (32.5%). 

### 3.2. Analgesic Activity

Compounds (**5–10**) have shown moderate analgesic activity (11.6–19.3%). The compound 9 showed good analgesic activity (19.3%) then compounds **5**, **6**, **7**, **8**, and 10. The formation of azetidinones (**11–16**) and thiazolidinones (**17–22**) from compounds (**5–10**) showed better analgesic activity than corresponding arylidene derivatives. Among these, compound 21 showed the better analgesic activity (29.6%) than other tested compounds. The results of the biological study are depicted in Table 1.

## 4. Conclusion

It may be concluded that the compounds having 4-chlorophenyl group of quinazolinone moiety showed better anti-inflammatory activity than phenyl moiety. Azetidinone and thiazolidinone derivatives were found active than corresponding schiff base derivatives. Further, thiazolidinones showed better anti-inflammatory and analgesics activities in comparison to their corresponding azetidinones. Among all the tested compounds, compound 21 showed better anti-inflammatory (32.5%) and analgesics (29.6%) activities than other compounds of series.

## Figures and Tables

**Table 1:** Anti-inflammatory and analgesic data of compounds **5**–**22** *. *

Comp. number	Dose (mg/kg p.o.)	Anti-inflammatory activity % oedema inhibition	Analgesic activity %
**5**	50	15.1	11.6
**6**	50	21.8	16.2
**7**	50	16.7	14.9
**8**	50	18.2	17.1
**9**	50	24.1	19.3
**10**	50	20.4	18.5
**11**	50	18.3	14.6
**12**	50	24.6	18.7
**13**	50	20.8	16.2
**14**	50	22.4	20.6
**15**	50	27.3	25.8
**16**	50	26.1	22.2
**17**	50	22.9	16.3
**18**	50	26.3	19.8
**19**	50	24.5	22.4
**20**	50	27.1	25.1
**21**	50	32.5	29.6
**22**	50	29.8	28.3
Phenylbutazone	50	38.8	36.5
